# Glucose Stimulation Induces Dynamic Change of Mitochondrial Morphology to Promote Insulin Secretion in the Insulinoma Cell Line INS-1E

**DOI:** 10.1371/journal.pone.0060810

**Published:** 2013-04-02

**Authors:** Bong Sook Jhun, Hakjoo Lee, Zheng-Gen Jin, Yisang Yoon

**Affiliations:** 1 Department of Physiology, Medical College of Georgia, Georgia Regents University, Augusta, Georgia, United States of America; 2 Center for Translational Medicine, Department of Medicine, Thomas Jefferson University, Philadelphia, Pennsylvania, United States of America; 3 Department of Medicine, University of Rochester School of Medicine and Dentistry, Rochester, New York, United States of America; 4 Aab Cardiovascular Research Institute, University of Rochester School of Medicine and Dentistry, Rochester, New York, United States of America; Boston University, United States of America

## Abstract

Fission and fusion of mitochondrial tubules are the major processes regulating mitochondrial morphology. However, the physiological significance of mitochondrial shape change is poorly understood. Glucose-stimulated insulin secretion (GSIS) in pancreatic β-cells requires mitochondrial ATP production which evokes Ca^2+^ influx through plasma membrane depolarization, triggering insulin vesicle exocytosis. Therefore, GSIS reflects mitochondrial function and can be used for evaluating functional changes associated with morphological alterations of mitochondria. Using the insulin-secreting cell line INS-1E, we found that glucose stimulation induced rapid mitochondrial shortening and recovery. Inhibition of mitochondrial fission through expression of the dominant-negative mutant DLP1-K38A eliminated this dynamic mitochondrial shape change and, importantly, blocked GSIS. We found that abolishing mitochondrial morphology change in glucose stimulation increased the mitochondrial inner membrane proton leak, and thus significantly diminished the mitochondrial ATP producing capacity in response to glucose stimulation. These results demonstrate that dynamic change of mitochondrial morphology is a previously unrecognized component for metabolism-secretion coupling of pancreatic β-cells by participating in efficient ATP production in response to elevated glucose levels.

## Introduction

Mitochondria are dynamic organelles, constantly changing their shape and size through fission and fusion. Dynamin-related large GTPases are the main components mediating mitochondrial fission and fusion. DLP1/Drp1 mediates mitochondrial fission whereas two mitofusin isoforms, Mfn1/Mfn2, and OPA1 are involved in fusion of the outer and inner mitochondrial membrane, respectively [1]–[5]. Fission and fusion events occur in a balanced frequency to maintain normal mitochondrial morphology. Mitochondrial fission and fusion have been implicated in preserving proper mitochondrial function. Disrupted mitochondrial morphologies are associated with numerous human disorders including neurodegeneration, cardiovascular disease, metabolic disease, and aging. Mutations in fission/fusion proteins resulting in hereditary diseases or lethal effect in humans indicate that disrupted mitochondrial morphology is causal for the harmful consequence presumably through mitochondrial dysfunction [6]–[9]. In addition, mitochondrial poisons causing mitochondrial dysfunction also induce disrupted mitochondrial morphology [10]–[13]. These observations suggest that mitochondrial morphology and function are closely linked and influence each other. However, mechanistic link of the mitochondrial form-function relationship and the physiological significance of mitochondrial shape change are poorly understood.

Pancreatic β-cells are glucose sensors that regulate body metabolism by secreting insulin. In response to elevated blood glucose levels, β-cells take up glucose and metabolize it through mitochondrial oxidative phosphorylation, which increases cellular ATP concentration. The increased cytosolic ATP/ADP ratio induces plasma membrane depolarization by inhibiting the ATP-sensitive K^+^ channel. Subsequently, Ca^2+^ influx through the voltage-dependent Ca^2+^ channel increases cytosolic Ca^2+^, which directly triggers insulin vesicle exocytosis [14], [15]. This series of events, glucose-stimulated insulin secretion (GSIS), requires glucose metabolism and mitochondrial ATP production and is also referred to as metabolism-secretion coupling of pancreatic β-cells.

It has been shown that mitochondria in primary β-cells and insulin-secreting cell lines form highly interconnected reticulum and are dynamic undergoing fission and fusion [16]–[20]. Pancreatic islets from human diabetic patients and diabetic animal models often contain swollen and shorter mitochondria [17], [21], [22], suggesting potential disruptions of mitochondrial fission/fusion in diabetic conditions. However, it is unclear whether this morphological change is causative, or is an effect from high glucose, cell injury, or additional factors in diabetic milieu. Primary β-cells cultured in the presence of high fat or high fat/high glucose displayed mitochondrial fragmentation and apoptosis, indicating a gluco-lipotoxic effect on mitochondrial morphology and cell function [20]. On the other hand, experimental perturbation of mitochondrial fission and fusion has been shown to affect GSIS [19], [23]–[25]. However, underlying mechanisms of how mitochondrial morphology and dynamics participate in GSIS are not understood.

In this study, we used the GSIS to examine the role of mitochondrial morphology in insulin secretion. Using the insulin-secreting rat insulinoma cell line INS-1E [26], we observed rapid mitochondrial shortening and recovery upon glucose stimulation. Inhibition of mitochondrial fission abolished this dynamic morphological change and blocked insulin secretion. We found that eliminating the glucose-induced mitochondrial morphology change decreased the mitochondrial ATP producing capacity in response to elevated glucose levels by increasing mitochondrial inner membrane proton leak. These results demonstrate that dynamic change of mitochondrial morphology is a new component of GSIS, controlling mitochondrial ATP production and thus insulin secretion in pancreatic β-cells.

## Results

### Insulin secretion upon glucose stimulation in INS-1E cells requires mitochondrial function and Ca^2+^ influx

**Figure 1:**
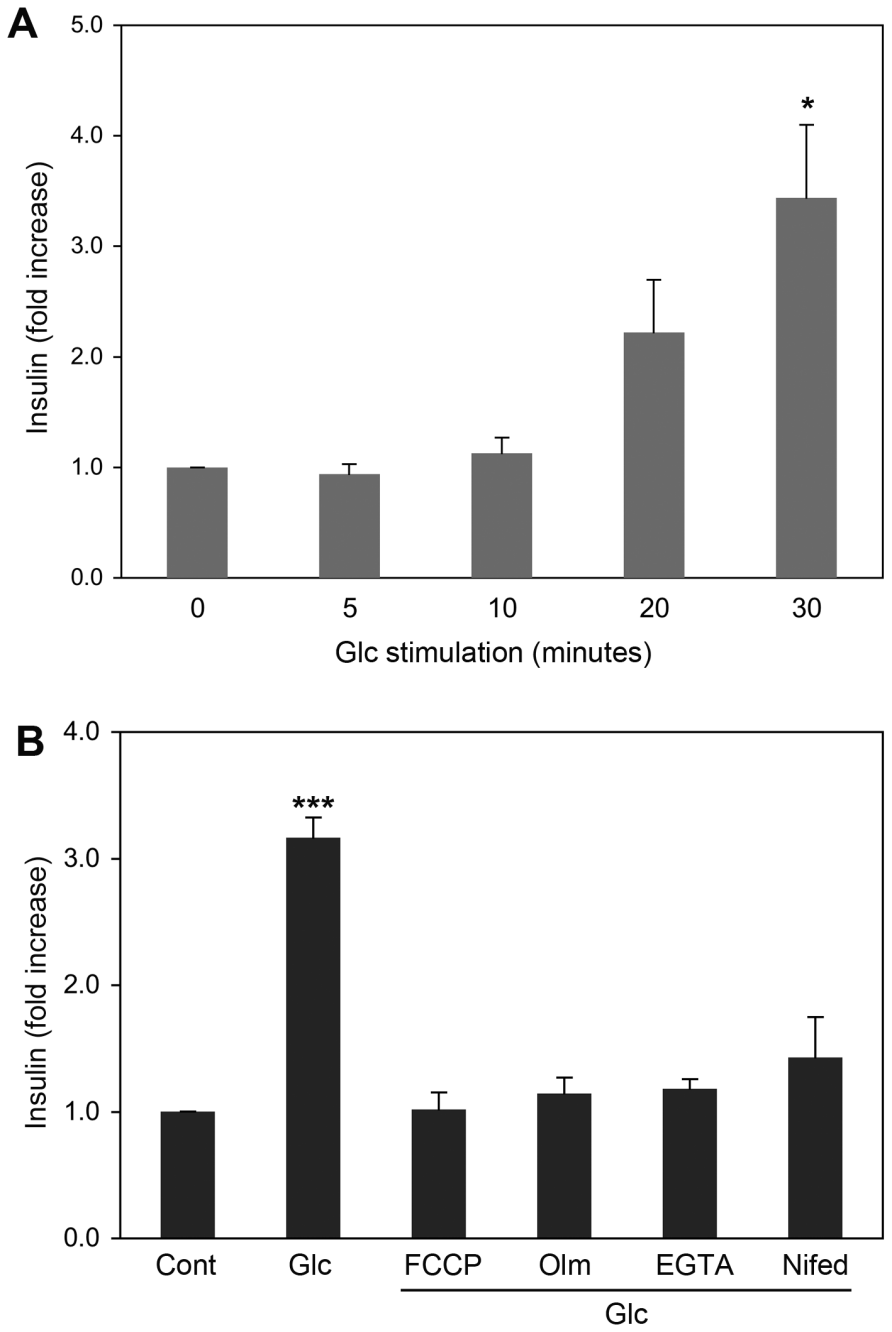
Glucose-stimulated insulin secretion requires mitochondrial function and Ca^2+^ influx. (A) Insulin secretion of INS-1E cells was measured at different times of 20 mM glucose stimulation. Secreted insulin levels were determined by ELISA. Significant increases of insulin were detected in 30-minute glucose stimulation. (B) Insulin secretion was assayed in INS-1E cells incubated in 20 mM glucose for 30 min in the presence of FCCP, oligomycin (Olm), EGTA, or Nifedipine. Error bars represent SEM. n = 3. * and *** are for *P*<0.05 and *P*<0.001, respectively, with no glucose control.

We evaluated GSIS in the insulin-secreting rat insulinoma cell line INS-1E. Stimulating cells with 20 mM glucose provoked insulin secretion. Measuring the insulin accumulation in the medium indicated a three to five fold increase by 30 minutes (Fig. 1A). Unlike the parental INS-1 line, INS-1E has been shown to have an optimal/maximal insulin secretion response at 15–20 mM glucose concentrations [26], [27]. Because 20 mM glucose can be considered high enough to induce glucotoxicity, we tested INS-1E cell death in 12 and 20 mM glucose incubations. INS-1E cells in 20 mM glucose showed no difference from those in 12 mM glucose and control incubations in cell viability up to 2 hours in trypan blue exclusion assays (Fig. S1A). Additionally, propidium iodide positive cells were less than 1% in all incubations with different glucose concentrations (Fig. S1B), indicating no increased glucotoxic effect with 20 mM glucose. Next, we tested the requirement of mitochondrial function for GSIS in this experimental system. Treating cells with the mitochondrial uncoupler FCCP abolished GSIS (Fig. 1B). In addition, the mitochondrial ATP synthase inhibitor oligomycin also blocked insulin secretion, indicating that mitochondrial ATP synthesis is necessary for GSIS (Fig. 1B). The penultimate step of the insulin secretion cascade is the Ca^2+^ influx induced by the plasma membrane depolarization. Nifedipine, a plasma membrane Ca^2+^ channel inhibitor, blocked GSIS and so did chelating the extracellular Ca^2+^ by EGTA (Fig. 1B). These results confirm previous findings [28]–[32] and demonstrate that insulin secretion upon glucose stimulation in INS-1E requires mitochondrial function and Ca^2+^ influx.

### Glucose stimulation induces reversible shortening of mitochondrial tubules

**Figure 2:**
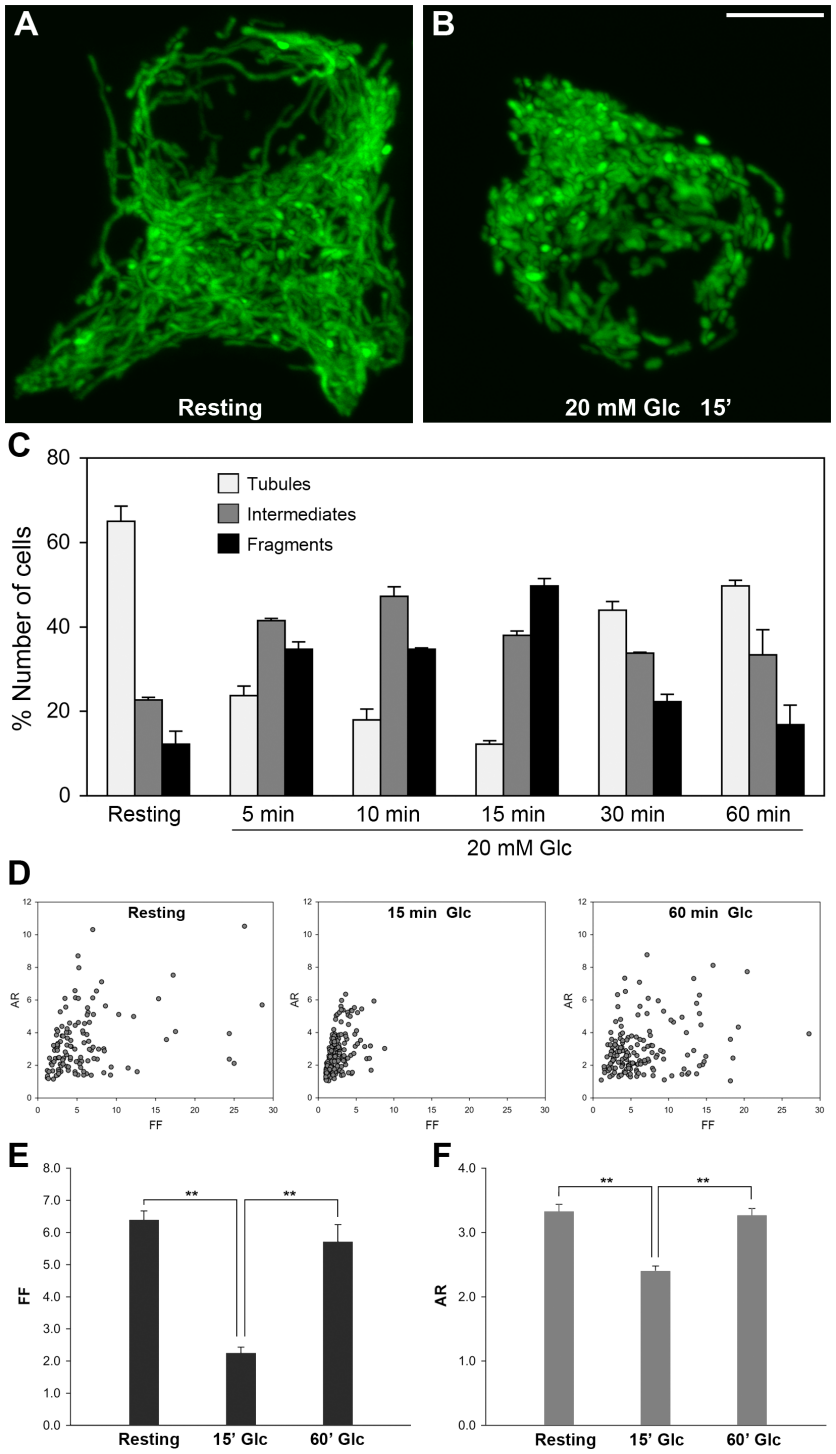
Glucose stimulation of INS-1E cells induces reversible shortening of mitochondrial tubules. (A, B) 3-D reconstructed confocal images of mitochondria showed tubular and reticular networks in resting conditions (A), and short and fragmented morphology upon 15-minute glucose stimulation (B). Scale bar, 5 µm. (C) Cell counting for different mitochondrial morphologies revealed that the glucose stimulation-induced mitochondrial shortening was reversible. The number of cells containing shorter mitochondria increased within 15 minutes of glucose stimulation and decreased at 30- and 60-minute incubations. (D-F) Mitochondrial shape analyses: individual mitochondria plotted for their FF and AR showed that many mitochondria in cells in a resting condition and following 60-minute glucose stimulation had higher values of FF and AR, whereas FF and AR values of mitochondria at 15 minutes of glucose stimulation were clustered with lower values (D). Average FF (E) and AR (F) values demonstrated that long mitochondrial tubules at resting conditions became short at 15-minute glucose stimulation and reverted back to elongated mitochondria at 60-minute incubation. Error bars are SEM. n = 6, 9, and 6 for resting, 15 and 60 minute glucose incubations, respectively. **, *P*<0.01.

As shown in Fig. 1, GSIS requires normal mitochondrial function. Because mitochondrial morphology is closely associated with functional states of mitochondria [33], we next examined the mitochondrial morphology in glucose stimulation. Mitochondrially targeted green fluorescent protein (mitoGFP) was expressed in INS-1E cells to visualize mitochondria. Three-dimensional reconstruction of confocal image slices showed that INS-1E cells in resting conditions contained tubular mitochondria forming network organization (Fig. 2A, Video S1). We found that, upon stimulation with 20 mM glucose, mitochondria rapidly became short and fragmented within 15 minutes (Fig. 2B, Video S2). Additionally, continuous incubation in the increased glucose concentration revealed that mitochondrial shape in INS-1E cells undergoes reversible changes with glucose stimulation. For quantification of this morphological change, different mitochondrial morphologies were categorized into ‘Tubules’ for long tubules of networks, ‘Fragments” for short tubules and small spheres, and ‘Intermediates’ for intermediate length of tubules or the mixture of tubules and fragments (Fig. S2). Cell counting at different times indicated that the mitochondrial shortening peaked around 15 minutes and long tubular mitochondria became prevalent again by 30 and 60 minutes of glucose stimulation (Fig. 2C). For more objective quantification, computer-assisted morphometric analyses were also performed to calculate form factor (FF) and aspect ratio (AR) of individual mitochondria that represent shape complexity and length [34]. INS-1E cells in resting conditions and glucose stimulation for 60 minutes contained numerous mitochondria with higher values of FF and AR, indicating that mitochondria are more elongated and branched (Fig. 2D). On the other hand, in cells incubated in 20 mM glucose for 15 minutes, both values become smaller, representing short and fragmented mitochondria (Fig. 2D). Average values of FF and AR from cells stimulated for 15 minutes were significantly lower than those from cells in resting conditions and 60-minute glucose stimulation (Fig. 2E, F). These results demonstrate that glucose stimulation of INS-1E cells induces rapid and reversible shortening of mitochondrial tubules.

### Inhibiting mitochondrial fission abolishes the glucose stimulation-induced dynamic change of mitochondrial morphology

**Figure 3:**
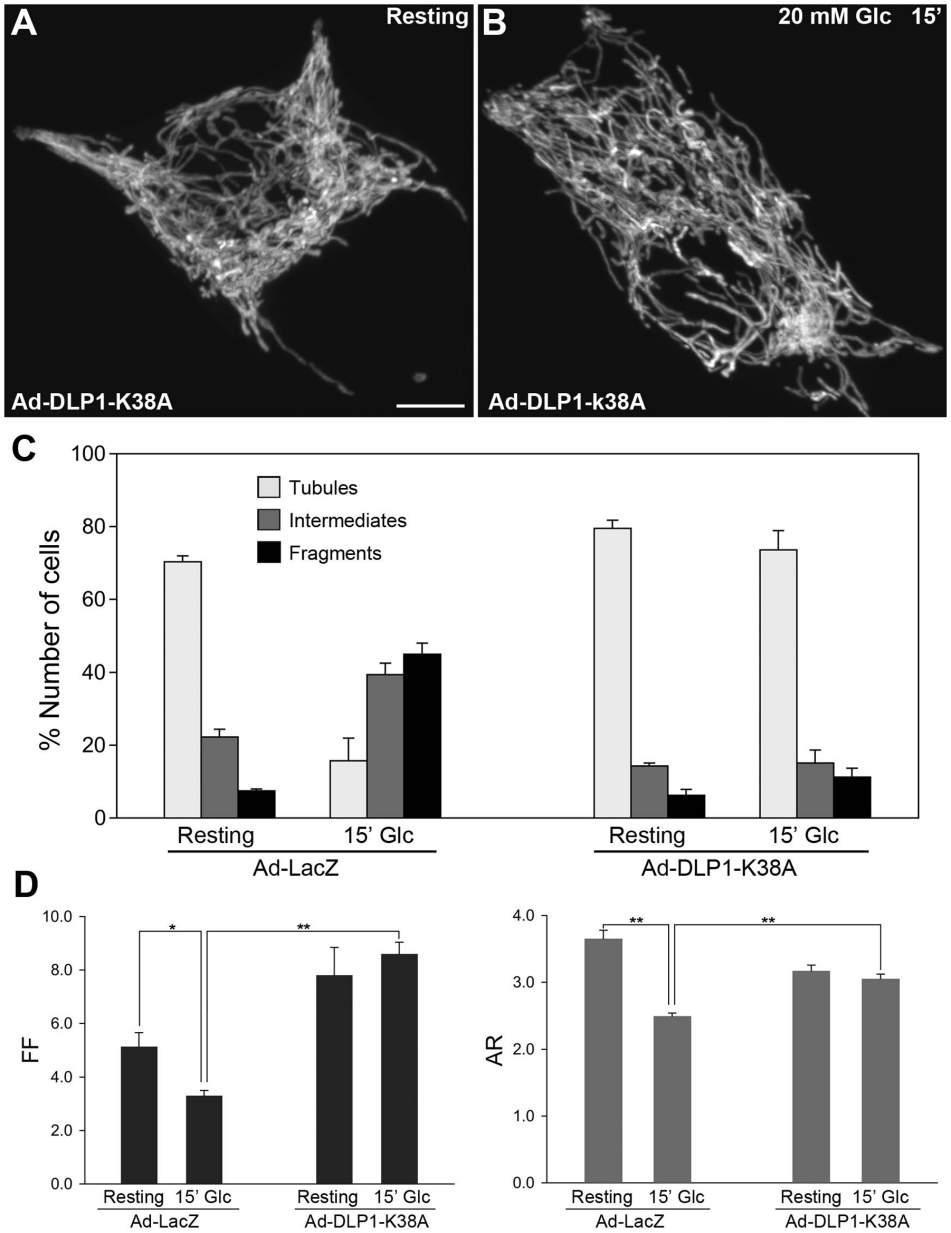
Inhibition of mitochondrial fission eliminates the glucose stimulation-induced reversible change of mitochondrial morphology. (A, B) Confocal images of mitochondria in resting (A) and glucose stimulated (B) conditions show long tubular morphology. Scale bar, 5 µm. (C) Cell counting showed that glucose stimulation did not change mitochondrial morphology when fission was inhibited (Ad-DLP1-K38A) whereas glucose stimulation-induced mitochondrial shortening was observed in control cells (Ad-LacZ). (D) Morphometric analyses show that fission inhibition prevented mitochondrial shortening upon glucose stimulation (Ad-DLP1-K38A). Error bars are SEM. n = 7 for Ad-LacZ resting and glucose, and Ad-DLP1-K38A resting, and n = 9 for Ad-DLP1-K38A glucose stimulation. *, *P*<0.05. **, *P*<0.01.

Prevalence of shorter mitochondria upon glucose stimulation raises a possibility that mitochondrial fission may play a role in insulin secretion. We tested whether mitochondrial fission is required for mitochondrial shortening and fragmentation observed in glucose stimulation. Adenovirus carrying the dominant-negative fission mutant DLP1-K38A (Ad-DLP1-K38A) was used to inhibit mitochondrial fission [35]. Immunofluorescence for DLP1 indicated that the adenoviral transduction resulted in expression of DLP1-K38A in all cells in culture by 48-hour post-infection, judged by characteristic bright aggregates formed within the cytoplasm [3], [36] (Fig. S3). We examined mitochondrial morphology in INS-1E cells co-infected with Ad-DLP1-K38A and Ad-mitoGFP. While mitochondrial hyperfusion upon fission inhibition was less appreciable in resting conditions due to highly interconnected networks of INS-1E mitochondria [16], [18], [19], we found that the long tubular mitochondrial morphology persisted in glucose stimulation (Fig. 3A and B, Videos S3 and S4). Both cell counting and morphometric analyses demonstrated a prevalence of long tubular mitochondria regardless of glucose stimulation in DLP1-K38A-expressing cells. Mitochondria in control cells infected with Ad-LacZ along with Ad-mitoGFP were short and fragmented at 15-minute glucose stimulation whereas those in Ad-DLP-K38A-infected cells maintained tubular mitochondria in glucose stimulation (Fig. 3C). Morphometric analyses also demonstrate the persistence of long tubular mitochondria in DLP1-K38A-expressing cells in glucose stimulation (Fig. 3D). In addition, increased values of FF by DLP1-K38A expression indicate that fission inhibition induced the formation of elongated tubules and network structure. A slight decrease of AR in DLP1-K38A cells suggested that mitochondrial tubules were longer and thus more curved, resulting in decreased maximal axis values in shape analyses. These data indicate that mitochondrial fission is necessary for mitochondrial shortening and that inhibiting fission eliminates the dynamic mitochondrial shape change in glucose stimulation.

### Inhibition of mitochondrial fission blocks glucose-stimulated insulin secretion but does not affect the KCl-induced insulin secretion

**Figure 4:**
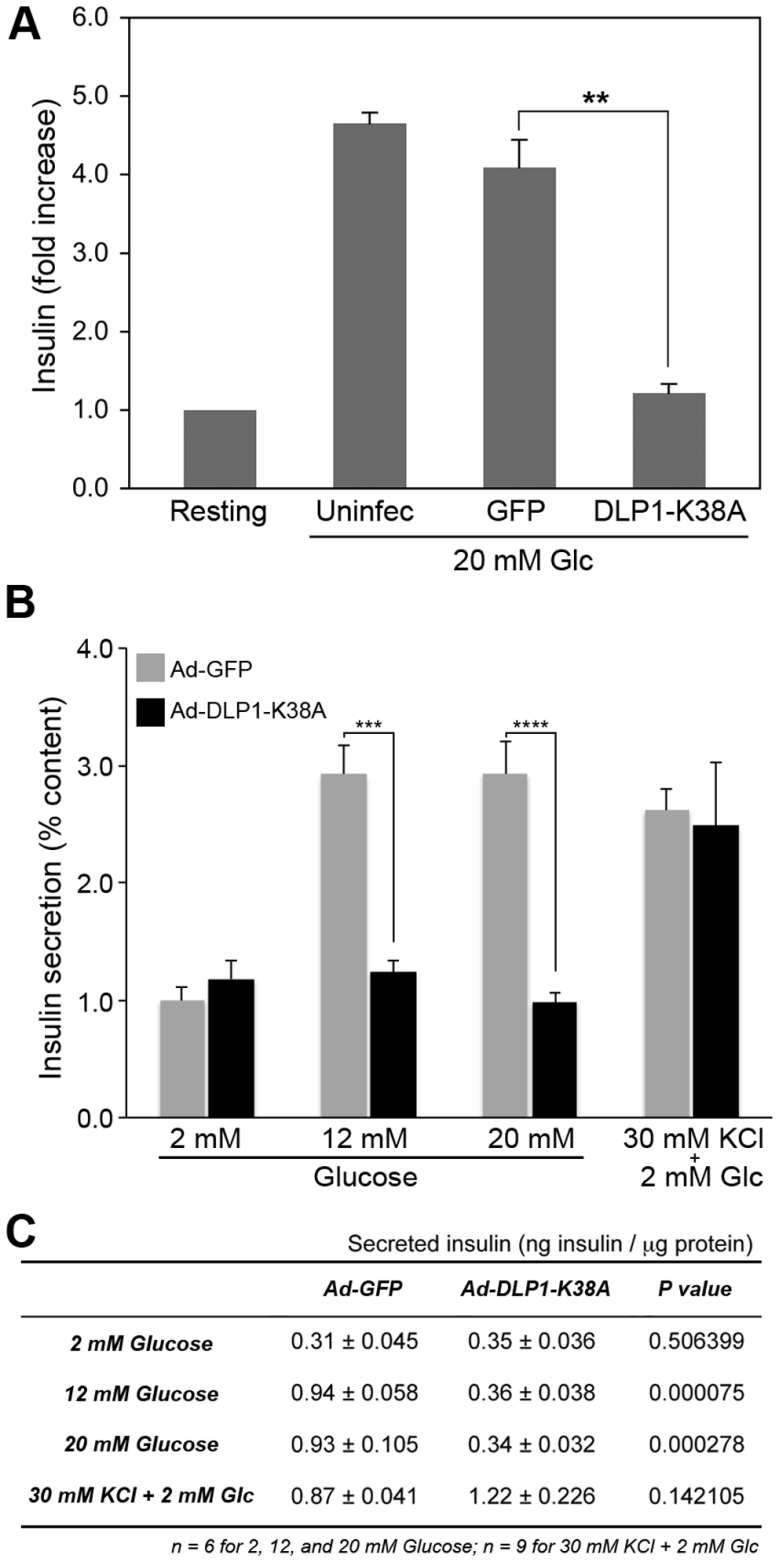
Inhibition of mitochondrial fission blocks the glucose-stimulated insulin secretion but does not affect the KCl-induced insulin secretion. (A) Glucose stimulation for 30 minutes induces insulin secretion in control cells (Uninfected and GFP) whereas fission inhibition (DLP1-K38A) decreased the secreted insulin level to that of resting conditions. (B) Insulin secretion was presented as % content, showing diminished levels of secreted insulin in cells infected with Ad-DLP1-K38A in stimulations with both 12 mM and 20 mM glucose. In contrast, KCl stimulation increased insulin secretion in both control and Ad-DLP1-K38A-infected cells. Error bars are SEM. n = 6 and 9 for glucose and KCl stimulations, respectively. ***, *P*<0.001 and ****, *P*<0.0001. (C) The amounts of secreted insulin normalized against total protein. There is no significant difference between control and fission inhibited cells in the amounts of secreted insulin at basal conditions (2 mM Glucose) and with KCl stimulation whereas DLP1-K38A-expressing cells show greatly decreased insulin levels with glucose stimulations.

To test whether the glucose-induced mitochondrial shape change plays a role in GSIS, we assessed the insulin secretion in INS-IE cells expressing DLP1-K38A. In control cells infected with adenovirus carrying green fluorescent protein (Ad-GFP), insulin secretion increased to a level indistinguishable from that in uninfected normal INS-1E cells (Fig. 4A). Remarkably, however, we consistently found that cells infected with Ad-DLP1-K38A showed little increase in the secreted insulin level in glucose stimulation (Fig. 4A). To substantiate this finding further, GSIS was tested with 12 mM and 20 mM glucose concentrations and expressed as % content (Fig. 4B). The amounts of secreted insulin in basal conditions showed no difference in both control and Ad-DLP1-K38A, indicating that fission inhibition does not affect basal insulin secretion (Fig. 4B,C). However, we found that cells expressing DLP1-K38A were unable to secrete insulin further in response to both 12 and 20 mM glucose stimulations (Fig. 4B,C). These results demonstrate that preventing glucose-induced mitochondrial shortening blocks insulin secretion, suggesting that the fission-mediated mitochondrial shape change plays an important role in the GSIS.

The metabolism-secretion coupling for GSIS requires the plasma membrane depolarization that induces the Ca^2+^ influx for insulin vesicle exocytosis. This mechanism was maintained in the INS-1E cell line as direct plasma membrane depolarization by KCl evoked insulin secretion in INS-1E cells (Fig. 4B). To determine where mitochondrial fission participates in the GSIS, we tested the effect of inhibiting mitochondrial fission on the KCl-induced insulin secretion. We found that KCl stimulation of INS-1E cells infected with Ad-DLP1-K38A still increased secreted insulin levels, demonstrating that the fission inhibition has little effect on insulin secretion upon KCl stimulation (Fig. 4B,C). These results indicate that fission inhibition affects neither plasma membrane depolarization nor insulin vesicle exocytosis, and suggest that impaired insulin secretion by fission inhibition is likely due to altering mitochondrial function.

### Eliminating dynamic mitochondrial shape change diminishes mitochondrial capacity to increase ATP production in glucose stimulation

**Figure 5:**
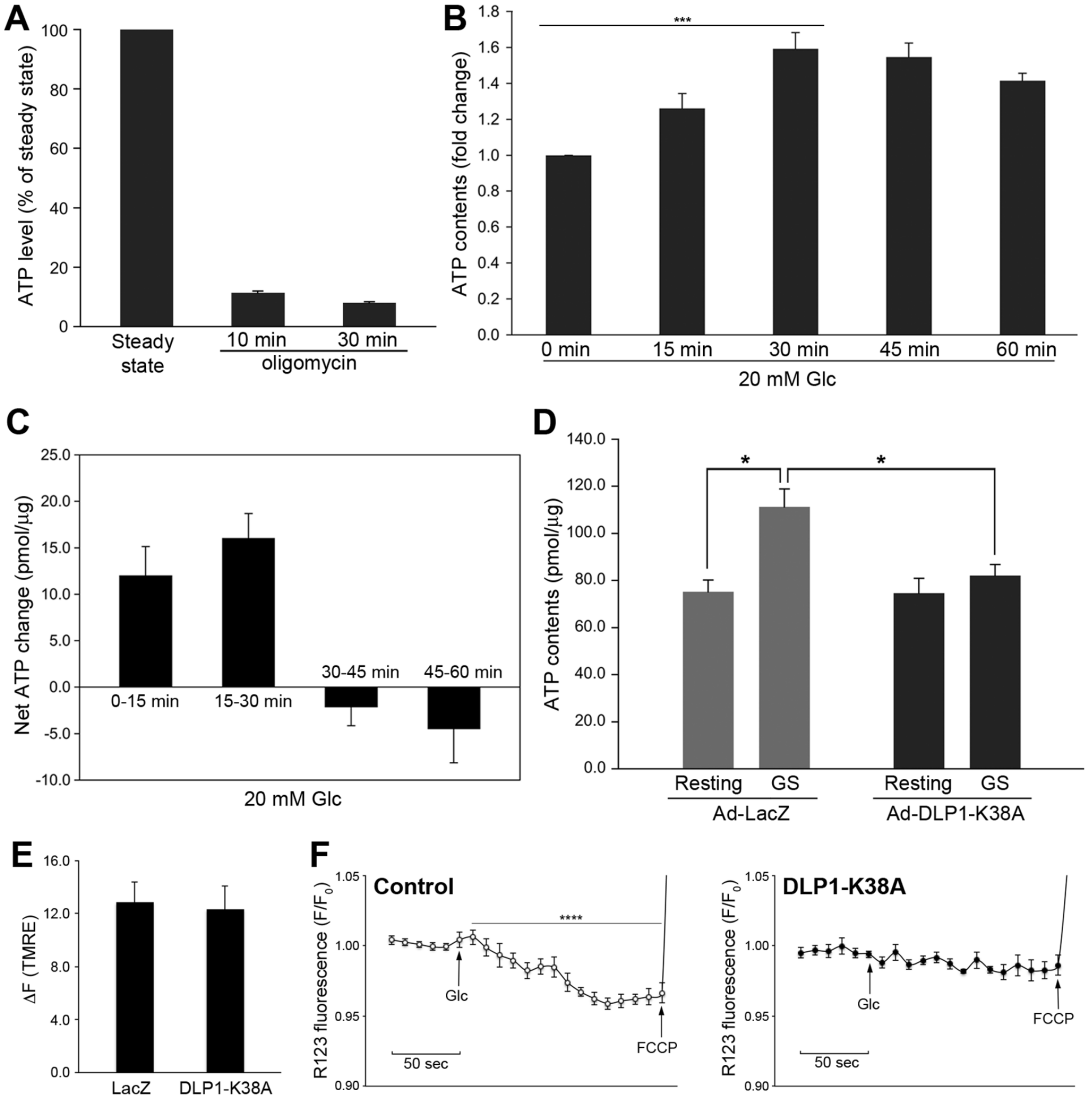
Fission inhibition limits mitochondrial ATP producing capacity as well as membrane potential increase in response to glucose stimulation. (A) Oligomycin treatment for 10 and 30 minutes drastically decreased the ATP contents in INS-1E cells. (B, C) ATP contents measured at different times of glucose stimulation showed that ATP increase was restricted to the first 30 minutes of the stimulation. Error bars are SEM. n = 6. ***, *P*<0.001 by one-way ANOVA. (D) Inhibition of mitochondrial fission (Ad-DLP1-K38A) prevented the glucose-stimulated ATP production whereas it did not affect ATP levels in resting conditions. ATP contents were measured at 30 minutes of glucose stimulation. GS, glucose stimulation. Error bars are SEM. n = 4. *, *P*<0.05. (E) Mitochondrial membrane potential assessed by TMRE at basal conditions. The difference in mean TMRE fluorescence measured before and after FCCP addition (ΔF_TMRE_) showed no difference in cells expressing LacZ and DLP1-K38A. Error bars are SEM. n = 25. (F) Glucose stimulation quenches Rhodamine123 fluorescence in control cells but not in DLP1-K38A-expressing cells. Fluorescence at every 10 seconds was normalized against the fluorescence value at basal conditions (F_0_) to express changes of fluorescence (F/F_0_). Control cells showed statistically significant decrease in rhodamine123 fluorescence upon 20 mM glucose addition (*P* = 0.00001 by one-way ANOVA) whereas no significance fluorescence decrease was observed in DLP1-K38A-expressing cells (*P* = 0.80566, one-way ANOVA). Error bars are SEM, n = 4.

Our results suggest that mitochondrial morphology change may play a role in ATP production by mitochondria during GSIS. We first measured cellular ATP levels during glucose stimulation in INS-1E cells. INS-1E cells produced ATP mainly from mitochondrial oxidative phosphorylation, as more than 90% of the total ATP contents were oligomycin-sensitive (Fig. 5A). ATP levels assayed at different times of continuous glucose stimulation showed a significant increase of ATP levels up to 30 minutes with a trend of decline afterward (Fig. 5B). Net ATP changes calculated for each 15-minute period indicated that the ATP increase occurred during the first 30 minutes of glucose stimulation and no further ATP increase was observed for the next 30-minute period (Fig. 5C). Notably, the approximate time frame for the increase and decrease in ATP levels during glucose stimulation appeared comparable with mitochondrial shortening and recovery that we observed (Fig. 2). To further investigate the role of mitochondrial fission in ATP production, we tested the effect of fission inhibition on cellular ATP levels. In resting conditions, there was no significant difference in cellular ATP levels between control and DLP1-K38A-expressing INS-1E cells. We found that glucose stimulation for 30 minutes increased the cellular ATP levels by approximately 50% in control cells. In cells expressing DLP1-K38A, however, this glucose-stimulated ATP increase was significantly suppressed (Fig. 5D). These data indicate that inhibition of mitochondrial fission causes a deficiency in mitochondrial ATP producing capacity in response to increased glucose levels.

We also measured the mitochondrial inner membrane potential in control and DLP1-K38A-expressing cells. In basal conditions, there was no difference in the inner membrane potential between LacZ- and DLP1-K38A-infected cells as measured by the potentiometric probe tetramethyl rhodamine ester (TMRE) (Fig. 5E). We also used the rhodamine123 fluorescence quenching method [26], [37] to test the effect of glucose stimulation on the mitochondrial membrane potential. Consistent with previous reports [26], we observed slight but statistically significant fluorescence quenching (∼5%) upon glucose stimulation in control cells, indicative of an increase of the inner membrane potential (Fig. 5F). Only a small increase of the membrane potential with glucose stimulation is likely due to the efficient coupling to ATP synthesis. In contrast, however, no decrease in fluorescence levels was found in cells expressing DLP1-K38A with glucose stimulation (Fig. 5F), suggesting that fission-deficiency causes a defect in enhancing the inner membrane potential in response to glucose stimulation. This observation is consistent with the finding that DLP1-K38A cells are unable to augment ATP levels in increased glucose concentrations. In addition, the lack of glucose-stimulated elevation of membrane potential in DLP1-K38A cells suggests that fission deficiency may cause alterations in the electron transport chain (ETC) activity.

### Inhibiting mitochondrial fission increases the mitochondrial inner membrane proton leak to limit the glucose-stimulated ATP enhancement

**Figure 6:**
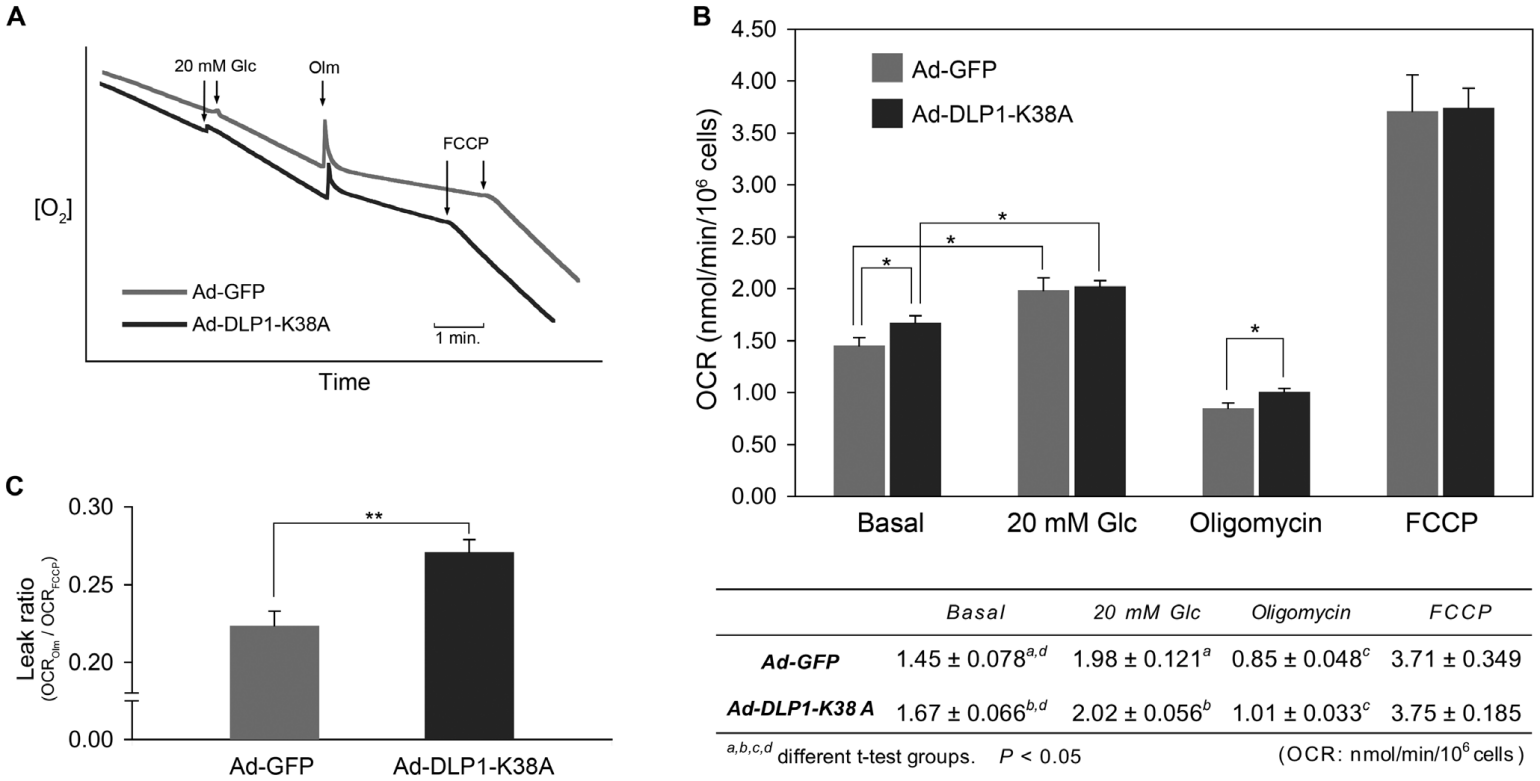
Inhibiting mitochondrial fission increases the mitochondrial inner membrane proton leak. (A) Oxygraphs showing oxygen consumption profiles of control (Ad-GFP) and fission inhibited (Ad-DLP1-K38A) cells. (B) Glucose stimulation increased the OCR in both control and fission inhibition. DLP1-K38A-expressing cells showed higher OCR in the presence of oligomycin. OCR values with different treatments are shown in the table. Error bars and ranges are SEM. n = 7. *, *P*<0.05. (C) Leak ratio was calculated as the proportion of the leak respiration (OCR_Olm_) out of the maximum leak respiration (OCR_FCCP_). Cells expressing DLP1-K38A had the significantly larger leak ratio compared to control cells. Error bars are SEM. n = 7. **, *P*<0.01.

To gain mechanistic insight as to how the inhibition of mitochondrial fission blocks the insulin secretion in glucose stimulation, we assessed the ETC activity by measuring cellular respiration. We found that increasing the glucose concentration from 2 to 20 mM enhanced the oxygen consumption rate (OCR) of INS-1E cells by 37% on average (Fig. 6A,B). This result indicates that the glucose stimulation accelerates respiration to increase the mitochondrial membrane potential and ATP production necessary for insulin secretion. Next, we measured respiration in DLP1-K38A-expressing INS-1E cells. We found that 20 mM glucose stimulation still increased the OCR in DLP1-K38A cells to the level similar to control (Fig. 6B), demonstrating that inhibiting mitochondrial fission did not affect the glucose-stimulated respiration increase. These data indicate that fission inhibition does not alter glucose metabolism and the reducing equivalent input into the ETC. Interestingly, we found that fission inhibition induced a small but statistically significant increase of the basal OCR (see Discussion). We further analyzed cellular respiration with oligomycin and the protonophore FCCP. Oligomycin blocks proton reentry through the F_0_F_1_-ATPase, and thus oxygen consumption in the presence oligomycin represents the mitochondrial inner membrane proton leak. In the presence of oligomycin, oxygen consumption rates were decreased in both control cells and cells expressing DLP1-K38A. However, DLP1-K38A-expressing cells revealed a substantially higher OCR than control cells in the presence of oligomycin (Fig. 6B). These data indicate that inhibition of mitochondrial fission increased the inner membrane proton leak. The maximum respiration rate of DLP1-K38A-expressing cells in the presence of FCCP showed no discernable difference from control cells, suggesting that the ETC of DLP1-K38A cells is functionally intact and maintains the normal capacity (Fig. 6A,B). Addition of antimycin A completely blocked oxygen consumption (OCR_AA_ = 0), indicating a negligible level of non-mitochondrial use of oxygen in INS-1E cells. The calculated leak ratio, the ratio of the leak rate to the maximum rate (OCR_Olm_/OCR_FCCP_), showed a significant increase of proton leak in cells expressing DLP1-K38A (Fig. 6C). An increased inner membrane proton leak in the metabolism-secretion coupling would result in futile proton pumping of the ETC without producing sufficient ATP. These results demonstrate that inhibition of mitochondrial fission induces the inner membrane proton leak, limits the glucose-stimulated ATP production, and blocks insulin secretion. The results described in this study indicate that dynamic change of mitochondrial morphology in glucose stimulation is necessary for insulin secretion activity of INS-1E cells by controlling mitochondrial ATP production.

## Discussion

Mitochondrial oxidative phosphorylation is a critical element for the metabolism-secretion coupling of pancreatic β-cells. In this study, we newly identified mitochondrial morphology change as previously unrecognized factor participating in GSIS. We observed mitochondrial shortening upon glucose stimulation through the fission process, and preventing this morphological change blocked GSIS. Interestingly, mitochondrial shortening was transient as mitochondria became elongated again by 30 minutes of glucose stimulation. GSIS has been shown to be biphasic in which the first phase occurs rapidly within 10 minutes by secreting the readily releasable pool of insulin vesicles at the plasma membrane whereas the second phase secretion involves new insulin synthesis and vesicle packaging through the secretory pathway [38], [39]. Although it is unclear whether the biphasic property of insulin secretion is preserved in INS-1E cells, rapid increase and decrease of mitochondrial shortening in glucose stimulation suggest a potential correlation of this morphological change with the rapid release of insulin vesicles. Nevertheless, cellular ATP levels calculated for 15-minute increments in the 60-minute glucose incubation revealed that glucose stimulation increases ATP production only within the first 30 minutes with little ATP increase in the next 30 minutes. This temporal profile of ATP levels in glucose stimulation correlates to some degree with the glucose-induced morphological change of mitochondria. These observations suggest that there is a close form-function relationship of mitochondria during GSIS and this morphological change may reflect the functional state of mitochondria. Previous ultrastructural observations of isolated mitochondria revealed that highly coupled state 3 mitochondria were more condensed and electron dense [40]. More recent electron tomographic analyses indicate that mitochondrial internal structure changes dynamically and is closely associated with functional states of mitochondria [41]. The mitochondrial morphology that we observed in glucose-stimulated INS-1E cells appeared tightly contracted, possibly representing energetically active, condensed mitochondria.

Recent studies indicate that elongated tubular mitochondria may produce more ATP as shown in cells under nutrient starvation, stress, or the S phase of the cell cycle [42]–[44]. On the other hand, our observations suggest that increased capacity of ATP production may be associated with short and condensed mitochondrial morphology in glucose stimulated INS-1E cells. However, the glucose-induced morphological change in INS-1E cells was a transient event in which long tubules become short, followed by recovery to elongated tubules within 30 minutes, the time frame in which the increased ATP production was observed (Fig. 2). Because two components of mitochondrial morphology, short and long tubules are both present in this time frame, it is difficult to conclude which mitochondrial form is representative of the more active state. Precise temporal analyses of mitochondrial morphology and function during early glucose stimulation will provide more information regarding the mitochondrial form-function relationship. Alternatively, it is also possible that mitochondrial morphology responds differently to different stimuli or stresses, producing similar energetic conditions under distinct morphologies.

An important finding in this study is that inhibition of mitochondrial fission blocks GSIS. It is possible that fission inhibition simply causes mitochondrial dysfunction and blocks GSIS. However, there are conflicting data about whether lack of fission alters mitochondrial function or not [45], [46]. It has been shown that mitochondrial fission is closely associated with mitochondrial quality control and inhibiting mitochondrial fission can accumulate dysfunctional mitochondria [23]. Indeed, downregulation of the fission protein Fis1 attenuated mitophagy and significantly decreased GSIS in rat insulinoma cells [23]. It cannot be ruled out that DLP1-K38A may decrease mitophagy and contribute to the diminished GSIS observed in the current study. In addition, other studies also showed that altered mitochondrial fission or fusion influenced GSIS [19], [24], [25]. Wollheim's group speculated that GSIS is related to cytoplasmic mitochondrial volume rather than specific morphologies [19]. While these studies clearly showed that altering mitochondrial morphology affects GSIS, our studies provide new mechanistic insight for the role of mitochondrial morphology in regulating insulin secretion in pancreatic β-cells. Our data show that fission inhibition in INS-1E cells has little effect on ATP production in resting conditions and the ETC capacity judged by the FCCP-induced maximum respiration, suggesting that ETC complex activities are largely normal in the fission inhibited condition. Further respiration analyses detected the increased inner membrane proton leak in fission inhibition. Interestingly, this amount of proton leak did not affect the basal level of ATP production, but only decreased the glucose-stimulated ATP production. Respiration data indicate that INS-1E mitochondria appear to have a relatively large reserve capacity, as less than a half of the maximal capacity is used at resting conditions. Because the detected increase of proton leak (20–30%) falls within the reserve capacity, it is predicted that this level of proton leak would be readily compensated and have a minimal effect on the ATP production in basal conditions. Indeed, we observed the increased basal respiration in fission inhibition, indicating that the steady-state ATP production is maintained through the compensatory respiration increase in resting conditions. Upon glucose stimulation, however, the ETC activity is pushed upward to utilize the reserve capacity and thus the effect of the proton leak is magnified, which limits the glucose-stimulated ATP production, resulting in decreased GSIS. Although less likely, it is also possible that fission inhibition may directly affect the ATP synthase activity, independent of proton leak, to decrease ATP production in glucose stimulation.

The dominant-negative DLP1-K38A inhibits mitochondrial fission and induces mitochondrial elongation, eliminating the dynamic change of mitochondrial morphology in glucose stimulation. We identified the increased inner membrane proton leak as a mechanism blocking GSIS in fission inhibition. These observations suggest that the glucose-induced change of mitochondrial morphology plays a role in controlling respiration coupling efficiency. How respiration coupling is controlled through the mitochondrial morphology change in glucose stimulation is an open question. Perhaps, normal mitochondrial dynamics in resting conditions represents a limited flux between partially coupled and partially uncoupled states. On the other hand, shortened and condensed mitochondria induced upon glucose stimulation could be regarded as tightly coupled mitochondria as suggested above. It has been speculated that mitochondrial morphology is related to respiratory complex organization and substrate channeling [10], [47]. Possibly, the glucose-induced mitochondrial morphology change may control the mitochondrial surface area to facilitate the metabolic intermediate input and, at the same time, increase the efficiency of the electron transport and the coupling to ATP synthesis by reorganizing respiratory complexes.

The current study identified dynamic change of mitochondrial morphology as a new component necessary for GSIS, providing further evidence for the close form-function relationship of mitochondria. Our data also provided mechanistic information that this morphological change of mitochondria plays a role in controlling respiration coupling, essential for insulin secretion in response to increased glucose levels. Many questions still remain. As discussed above, precise temporal resolution for mitochondrial morphology change, ATP production, and insulin secretion would provide useful information for how these components interface one another in GSIS. Altered respiration coupling in fission inhibition also provides a new insight for a mechanistic link contributing to the mitochondrial form-function correlation. Identifying the mechanisms of how mitochondrial shape change participates in regulating the respiration coupling will be important in understanding GSIS and the pathology of diabetes.

## Materials and Methods

### Cell culture

INS-1E cells [26], a well characterized clonal line derived from rat insulinoma INS-1 cells, were cultured in complete medium composed of RPMI 1640 supplemented with 5% heat-inactivated fetal bovine serum, 1 mM sodium pyruvate, 50 µM 2-mercaptoethanol, 2 mM glutamine, 10 mM HEPES, 100 U/ml penicillin, and 100 µg/ml streptomycin at 37°C in a humidified atmosphere containing 5% CO_2_ as described [26]. For adenoviral infection, INS-1E cells seeded and cultured for 2–3 days were infected with appropriate adenovirus vectors. At 6 hours after virus addition, cells were rinsed and further cultured in fresh media. Adenovirus titers were obtained by an ELISA kit (Cell Biolabs, Inc.) that detects the adenovirus Hexon protein in HEK293 cells. The titer of Ad-DLP1-K38A was 2.5×10^10^ ifu/ml. Typically, adenoviruses were infected at MOI of 10.

### Insulin secretion assay

Before cell stimulation, INS-1E cells were incubated in serum free culture medium containing 2 mM glucose for 2 hours at 37 °C. Then, cells were washed and incubated with Krebs-Ringer bicarbonate HEPES buffer (KRBH, 135 mM NaCl, 3.6 mM KCl, 5 mM NaHCO_3_, 0.5 mM NaH_2_PO_4_, 0.5 mM MgCl_2_, 1.5 mM CaCl_2_, and 10 mM HEPES, pH 7.4, and 0.1% bovine serum albumin) plus 2 mM glucose for 30 min at 37°C. Cells were then stimulated with 12 mM glucose, 20 mM glucose or 30 mM KCl in KRBH buffer for indicated time. Where appropriate, cells were pretreated for 10 minutes with FCCP (1 µM), oligomycin (10 µM), EGTA (5 mM), or nifedipine (10 µM) prior to glucose stimulation. At the end of stimulation, the medium was collected and cleared by centrifugation. Supernatants were assayed for insulin contents using Mercodia rat insulin ELISA (Mercodia, Uppsala, Sweden). Secreted insulin levels were presented as % content or normalized against total cellular protein concentration.

### Mitochondrial morphology analyses

INS-1E cells expressing mitochondrially targeted GFP were fixed in 4% paraformaldehyde for 20 minutes at room temperature. When necessary, cells were permeabilized with 0.1% Triton X-100 and immunostained for DLP1 using rabbit anti-DLP1 antibodies and anti-rabbit Alexa 594 antibodies. Cells were observed and analyzed using Olympus FV1000 laser scanning confocal microscope or epifluorescence microscope (Olympus IX71). Mitochondrial morphologies were categorized into ‘Tubules’, ‘Intermediates’, or ‘Fragments’, and cells containing these morphologies were counted and expressed as a percent of the total cell count. More than 200 cells were counted for each counting and the experiments were repeated 3–6 times. For computer-assisted morphometric analyses, equalized fluorescent intensity representing mitochondria were acquired using a convolve filter through the NIH-developed ImageJ software (Wayne Rasband, NIH). After threshold, individual mitochondria were analyzed for form factor (FF: the reciprocal of circularity value) and aspect ratio (AR: major axis/minor axis) [34]. Both parameters have a minimal value of 1 when it is a perfect circle and increase as mitochondrial shape becomes long and complex.

### Cellular ATP measurement

Total cellular ATP concentrations were determined using an ATPLite™ luminescence assay system according to the manufacturer's instructions (PerkinElmer) and normalized by total protein concentration. Cells were treated with oligomycin (10 µM) to assess mitochondrial ATP production.

### Membrane potential measurements

The mitochondrial inner membrane potential was assessed using TMRE and Rhodamine123. Cells were incubated in the medium containing 2 mM glucose for 1 hour prior to loading with TMRE (50 nM) or Rhodamine123 (10 µM) for 20 minutes. TMRE fluorescence was imaged in KRBH plus 2 mM glucose using a rhodamine filter set by fluorescence microscopy. The inner membrane potential was presented as a difference of mean fluorescence intensity measured before and after FCCP addition (ΔF_TMRE_). To detect fluorescence quenching/dequenching, cells loaded with Rhodamine123 were stirred in KRBH plus 2 mM glucose in a cuvette, and fluorescence emission at 535 nm was measured with 480 nm excitation in sequential additions of glucose (20 mM) and FCCP (5 µM) (Black-Comet Spectrometer, StellarNet, Inc.).

### Respiration measurement

To determine cellular respiration, oxygen consumption rates were measured in whole cells by using a Clark-type O_2_ electrode. Cells expressing GFP or DLP1-K38A were stirred in a sealed chamber at 37°C in respiration medium (KRBH plus 2 mM glucose) and the decrease of the O_2_ concentration was measured as cellular oxygen consumption. Once constant rate was reached in basal conditions (2 mM glucose), glucose was added to a final concentration of 20 mM to measure the glucose-stimulated respiration. Oligomycin was added to measure oxygen consumption in the absence of the ATP synthase activity and FCCP to assess maximal respiration. For titration, OCR was measured with different concentrations of oligomycin and FCCP (Fig. S4). OCR was normalized by the number of cells in the chamber.

### Statistical analyses

Error bars in all graphs represent standard error of the mean (SEM). Two-tailed unpaired *t*-test was used to compare the two groups and one-way ANOVA to compare multiple groups. The *P* value was calculated with Microsoft Excel and StatPlus. *P*<0.05 was considered as statistically significant.

## Supporting Information

Figure S1 INS-1E cell viability assessment in different glucose concentrations. (A) Trypan blue exclusion assays of INS-1E cells incubated in 2, 12, and 20 mM glucose concentrations for 0.5, 1, and 2 hours showed more than 96% cell viability in all incubation conditions (*P* = 0.75575 by one-way ANOVA). (B) Propidium iodide staining of cells incubated in the same conditions as (A) showed less than 1% of dead cells in all incubations (*P* = 0.40877 by one-way ANOVA).
(TIF)

Figure S2 Mitochondrial morphologies categorized in INS-1E cells. INS-1E cells containing long tubules of networks (A; ‘Tubules’), short tubules and small spheres (B; ‘Fragments’), and intermediate tubule length or the mixture of tubules and fragments (C; ‘Intermediates’). Bottom panels are enlarged images of boxed regions.
(TIF)

Figure S3 DLP1-K38A expression. The dominant-negative DLP1-K38A mutant was expressed in INS-1E cells for 48 hours by adenoviral infection. Endogenous DLP1 appeared diffuse in the cytosol in this magnification (A). Overexpression of DLP1-K38A induced the formation of bright DLP1-containing aggregates in the cytoplasm (B). Inset shows the DLP1 immunoblot of cell lysates from control and DLP1-K38A-expressing cells. The molecular weight of the overexpressed DLP1-K38A protein is higher due to the use of a larger spliced variant for mutagenesis.
(TIF)

Figure S4 Titration of oligomycin and FCCP. OCR was measured with INS-1E cells in different concentrations of oligomycin (A) and FCCP (B). Concentrations higher than 1–2 µM showed maximal efficacy in both oligomycin and FCCP for decreasing and increasing the OCR, respectively.
(TIF)

Video S1 Confocal 3-D reconstruction for mitochondria in INS-1E in resting conditions
(AVI)

Video S2 Confocal 3-D reconstruction for mitochondria in INS-1E at 15 minutes of glucose stimulation
(AVI)

Video S3 Confocal 3-D reconstruction for mitochondria in INS-1E expressing DLP1-K38A in resting conditions
(AVI)

Videos S4 Confocal 3-D reconstruction for mitochondria in INS-1E expressing DLP1-K38A at 15 minutes of glucose stimulation
(AVI)

## References

[1] Santel A, Fuller MT (2001) Control of mitochondrial morphology by a human mitofusin. J Cell Sci 114: 867–874. 11181170 10.1242/jcs.114.5.867

[2] Smirnova E, Griparic L, Shurland DL, van der Bliek AM (2001) Dynamin-related protein Drp1 is required for mitochondrial division in mammalian cells. Mol Biol Cell 12: 2245–2256. 11514614 10.1091/mbc.12.8.2245 PMC58592

[3] Pitts KR, Yoon Y, Krueger EW, McNiven MA (1999) The dynamin-like protein DLP1 is essential for normal distribution and morphology of the endoplasmic reticulum and mitochondria in mammalian cells. Mol Biol Cell 10: 4403–4417. 10588666 10.1091/mbc.10.12.4403 PMC25766

[4] Cipolat S, Martins de Brito O, Dal Zilio B, Scorrano L (2004) OPA1 requires mitofusin 1 to promote mitochondrial fusion. Proc Natl Acad Sci U S A 101: 15927–15932. 15509649 10.1073/pnas.0407043101 PMC528769

[5] Yoon Y, Galloway CA, Jhun BS, Yu T (2011) Mitochondrial dynamics in diabetes. Antioxid Redox Signal 14: 439–457. 20518704 10.1089/ars.2010.3286 PMC3025181

[6] Alexander C, Votruba M, Pesch UE, Thiselton DL, Mayer S, et al (2000) OPA1, encoding a dynamin-related GTPase, is mutated in autosomal dominant optic atrophy linked to chromosome 3q28. Nat Genet 26: 211–215. 11017080 10.1038/79944

[7] Delettre C, Lenaers G, Griffoin JM, Gigarel N, Lorenzo C, et al (2000) Nuclear gene OPA1, encoding a mitochondrial dynamin-related protein, is mutated in dominant optic atrophy. Nat Genet 26: 207–210. 11017079 10.1038/79936

[8] Zuchner S, Mersiyanova IV, Muglia M, Bissar-Tadmouri N, Rochelle J, et al (2004) Mutations in the mitochondrial GTPase mitofusin 2 cause Charcot-Marie-Tooth neuropathy type 2A. Nat Genet 36: 449–451. 15064763 10.1038/ng1341

[9] Waterham HR, Koster J, van Roermund CW, Mooyer PA, Wanders RJ, et al (2007) A lethal defect of mitochondrial and peroxisomal fission. N Engl J Med 356: 1736–1741. 17460227 10.1056/NEJMoa064436

[10] Benard G, Bellance N, James D, Parrone P, Fernandez H, et al (2007) Mitochondrial bioenergetics and structural network organization. J Cell Sci 120: 838–848. 17298981 10.1242/jcs.03381

[11] De Vos KJ, Allan VJ, Grierson AJ, Sheetz MP (2005) Mitochondrial function and actin regulate dynamin-related protein 1-dependent mitochondrial fission. Curr Biol 15: 678–683. 15823542 10.1016/j.cub.2005.02.064

[12] Koopman WJ, Visch HJ, Verkaart S, van den Heuvel LW, Smeitink JA, et al (2005) Mitochondrial network complexity and pathological decrease in complex I activity are tightly correlated in isolated human complex I deficiency. Am J Physiol Cell Physiol 289: C881–890. 15901599 10.1152/ajpcell.00104.2005

[13] Legros F, Lombes A, Frachon P, Rojo M (2002) Mitochondrial fusion in human cells is efficient, requires the inner membrane potential, and is mediated by mitofusins. Mol Biol Cell 13: 4343–4354. 12475957 10.1091/mbc.E02-06-0330 PMC138638

[14] Kennedy ED, Maechler P, Wollheim CB (1998) Effects of depletion of mitochondrial DNA in metabolism secretion coupling in INS-1 cells. Diabetes 47: 374–380. 9519742 10.2337/diabetes.47.3.374

[15] Maechler P, Wollheim CB (1998) Role of mitochondria in metabolism-secretion coupling of insulin release in the pancreatic beta-cell. Biofactors 8: 255–262. 9914827 10.1002/biof.5520080313

[16] Plecita-Hlavata L, Lessard M, Santorova J, Bewersdorf J, Jezek P (2008) Mitochondrial oxidative phosphorylation and energetic status are reflected by morphology of mitochondrial network in INS-1E and HEP-G2 cells viewed by 4Pi microscopy. Biochim Biophys Acta 1777: 834–846. 18452700 10.1016/j.bbabio.2008.04.002

[17] Bindokas VP, Kuznetsov A, Sreenan S, Polonsky KS, Roe MW, et al (2003) Visualizing superoxide production in normal and diabetic rat islets of Langerhans. J Biol Chem 278: 9796–9801. 12514170 10.1074/jbc.M206913200

[18] Dlaskova A, Spacek T, Santorova J, Plecita-Hlavata L, Berkova Z, et al.. (2010) 4Pi microscopy reveals an impaired three-dimensional mitochondrial network of pancreatic islet beta-cells, an experimental model of type-2 diabetes. Biochim Biophys Acta. 10.1016/j.bbabio.2010.02.003 20144584

[19] Park KS, Wiederkehr A, Kirkpatrick C, Mattenberger Y, Martinou JC, et al (2008) Selective actions of mitochondrial fission/fusion genes on metabolism-secretion coupling in insulin-releasing cells. J Biol Chem 283: 33347–33356. 18832378 10.1074/jbc.M806251200 PMC2662262

[20] Molina AJ, Wikstrom JD, Stiles L, Las G, Mohamed H, et al (2009) Mitochondrial networking protects beta-cells from nutrient-induced apoptosis. Diabetes 58: 2303–2315. 19581419 10.2337/db07-1781 PMC2750232

[21] Anello M, Lupi R, Spampinato D, Piro S, Masini M, et al (2005) Functional and morphological alterations of mitochondria in pancreatic beta cells from type 2 diabetic patients. Diabetologia 48: 282–289. 15654602 10.1007/s00125-004-1627-9

[22] Deng S, Vatamaniuk M, Huang X, Doliba N, Lian MM, et al (2004) Structural and functional abnormalities in the islets isolated from type 2 diabetic subjects. Diabetes 53: 624–632. 14988246 10.2337/diabetes.53.3.624

[23] Twig G, Elorza A, Molina AJ, Mohamed H, Wikstrom JD, et al (2008) Fission and selective fusion govern mitochondrial segregation and elimination by autophagy. Embo J 27: 433–446. 18200046 10.1038/sj.emboj.7601963 PMC2234339

[24] Zhang Z, Wakabayashi N, Wakabayashi J, Tamura Y, Song WJ, et al (2011) The dynamin-related GTPase Opa1 is required for glucose-stimulated ATP production in pancreatic beta cells. Mol Biol Cell 22: 2235–2245. 21551073 10.1091/mbc.E10-12-0933 PMC3128526

[25] Men X, Wang H, Li M, Cai H, Xu S, et al (2009) Dynamin-related protein 1 mediates high glucose induced pancreatic beta cell apoptosis. Int J Biochem Cell Biol 41: 879–890. 18805504 10.1016/j.biocel.2008.08.031

[26] Merglen A, Theander S, Rubi B, Chaffard G, Wollheim CB, et al (2004) Glucose sensitivity and metabolism-secretion coupling studied during two-year continuous culture in INS-1E insulinoma cells. Endocrinology 145: 667–678. 14592952 10.1210/en.2003-1099

[27] Asfari M, Janjic D, Meda P, Li G, Halban PA, et al (1992) Establishment of 2-mercaptoethanol-dependent differentiated insulin-secreting cell lines. Endocrinology 130: 167–178. 1370150 10.1210/endo.130.1.1370150

[28] Morgan NG, Short CD, Rumford GM, Montague W (1985) Effects of the calcium-channel agonist CGP 28392 on insulin secretion from isolated rat islets of Langerhans. Biochem J 231: 629–634. 2934056 10.1042/bj2310629 PMC1152795

[29] Somers G, Devis G, Malaisse WJ (1979) Calcium antagonists and islet function. IX. Is extracellular calcium required for insulin release? Acta Diabetol Lat 16: 9–18. 377879 10.1007/BF02590758

[30] MacDonald MJ, Fahien LA (1990) Insulin release in pancreatic islets by a glycolytic and a Krebs cycle intermediate: contrasting patterns of glyceraldehyde phosphate and succinate. Arch Biochem Biophys 279: 104–108. 2186702 10.1016/0003-9861(90)90468-e

[31] McCormack JG, Longo EA, Corkey BE (1990) Glucose-induced activation of pyruvate dehydrogenase in isolated rat pancreatic islets. Biochem J 267: 527–530. 2185742 10.1042/bj2670527 PMC1131320

[32] Kennedy ED, Maechler P, Wollheim CB (1998) Effects of depletion of mitochondrial DNA in metabolism secretion coupling in INS-1 cells. Diabetes 47: 374–380. 9519742 10.2337/diabetes.47.3.374

[33] Chen H, Chan DC (2005) Emerging functions of mammalian mitochondrial fusion and fission. Hum Mol Genet 14: R283–R289. 16244327 10.1093/hmg/ddi270

[34] Koopman WJ, Verkaart S, Visch HJ, van der Westhuizen FH, Murphy MP, et al (2005) Inhibition of complex I of the electron transport chain causes O2^-^. -mediated mitochondrial outgrowth. Am J Physiol Cell Physiol 288: C1440–C1450. 15647387 10.1152/ajpcell.00607.2004

[35] Yu T, Sheu SS, Robotham JL, Yoon Y (2008) Mitochondrial fission mediates high glucose-induced cell death through elevated production of reactive oxygen species. Cardiovasc Res 79: 341–351. 18440987 10.1093/cvr/cvn104 PMC2646899

[36] Yoon Y, Pitts KR, McNiven MA (2001) Mammalian dynamin-like protein DLP1 tubulates membranes. Mol Biol Cell 12: 2894–2905. 11553726 10.1091/mbc.12.9.2894 PMC59722

[37] Perry SW, Norman JP, Barbieri J, Brown EB, Gelbard HA (2011) Mitochondrial membrane potential probes and the proton gradient: a practical usage guide. Biotechniques 50: 98–115. 21486251 10.2144/000113610 PMC3115691

[38] Nesher R, Cerasi E (2002) Modeling phasic insulin release: immediate and time-dependent effects of glucose. Diabetes 51 Suppl 1S53–59. 11815459 10.2337/diabetes.51.2007.s53

[39] Rorsman P, Eliasson L, Renstrom E, Gromada J, Barg S, et al (2000) The Cell Physiology of Biphasic Insulin Secretion. News Physiol Sci 15: 72–77. 11390882 10.1152/physiologyonline.2000.15.2.72

[40] Hackenbrock CR (1966) Ultrastructural bases for metabolically linked mechanical activity in mitochondria: I. Reversible ultrastructural changes with change in metabolic steady state in isolated liver mitochondria. J Cell Biol 30: 269–297. 5968972 10.1083/jcb.30.2.269 PMC2107001

[41] Mannella CA (2006) Structure and dynamics of the mitochondrial inner membrane cristae. Biochim Biophys Acta 1763: 542–548. 16730811 10.1016/j.bbamcr.2006.04.006

[42] Gomes LC, Di Benedetto G, Scorrano L (2011) During autophagy mitochondria elongate, are spared from degradation and sustain cell viability. Nat Cell Biol 13: 589–598. 21478857 10.1038/ncb2220 PMC3088644

[43] Tondera D, Grandemange S, Jourdain A, Karbowski M, Mattenberger Y, et al (2009) SLP-2 is required for stress-induced mitochondrial hyperfusion. Embo J 28: 1589–1600. 19360003 10.1038/emboj.2009.89 PMC2693158

[44] Mitra K, Wunder C, Roysam B, Lin G, Lippincott-Schwartz J (2009) A hyperfused mitochondrial state achieved at G1-S regulates cyclin E buildup and entry into S phase. Proc Natl Acad Sci U S A 106: 11960–11965. 19617534 10.1073/pnas.0904875106 PMC2710990

[45] Ishihara N, Nomura M, Jofuku A, Kato H, Suzuki SO, et al (2009) Mitochondrial fission factor Drp1 is essential for embryonic development and synapse formation in mice. Nat Cell Biol 11: 958–966. 19578372 10.1038/ncb1907

[46] Parone PA, Da Cruz S, Tondera D, Mattenberger Y, James DI, et al (2008) Preventing mitochondrial fission impairs mitochondrial function and leads to loss of mitochondrial DNA. PLoS ONE 3: e3257. 18806874 10.1371/journal.pone.0003257 PMC2532749

[47] Benard G, Rossignol R (2008) Ultrastructure of the mitochondrion and its bearing on function and bioenergetics. Antioxid Redox Signal 10: 1313–1342. 18435594 10.1089/ars.2007.2000

