# Supplementary figures and images for: Glucose Stimulation Induces Dynamic Change of Mitochondrial Morphology to Promote Insulin Secretion in the Insulinoma Cell Line INS-1E
Source: PLoS One. 2013 Apr 2;8(4):e60810. doi: 10.1371/journal.pone.0060810 (PMC3614983; doi:10.1371/journal.pone.0060810)

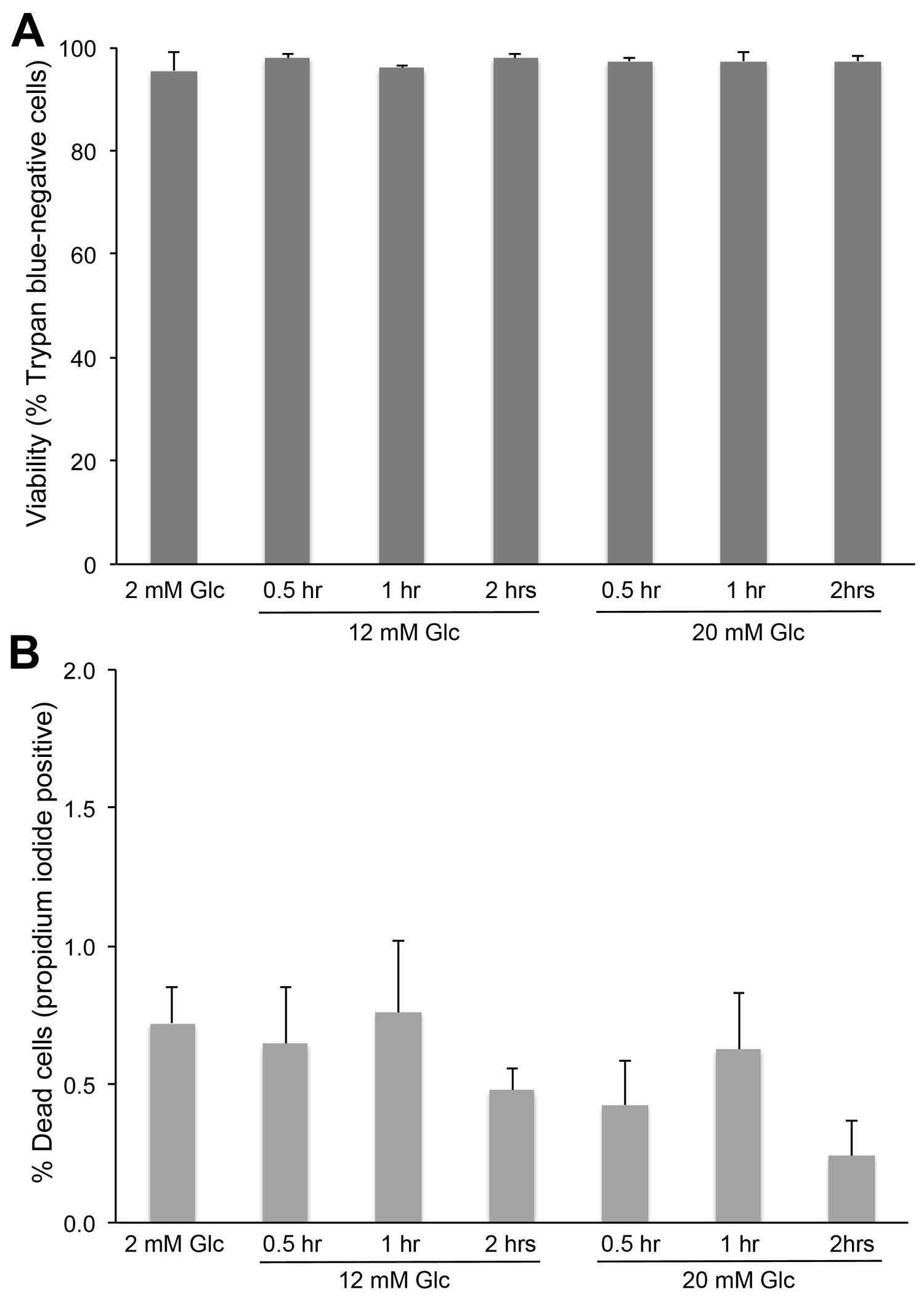

Supplement: Figure S1 — INS-1E cell viability assessment in different glucose concentrations. (A) Trypan blue exclusion assays of INS-1E cells incubated in 2, 12, and 20 mM glucose concentrations for 0.5, 1, and 2 hours showed more than 96% cell viability in all incubation conditions (P = 0.75575 by one-way ANOVA). (B) Propidium iodide staining of cells incubated in the same conditions as (A) showed less than 1% of dead cells in all incubations (P = 0.40877 by one-way ANOVA). (TIF) [file pone.0060810.s001.tif]

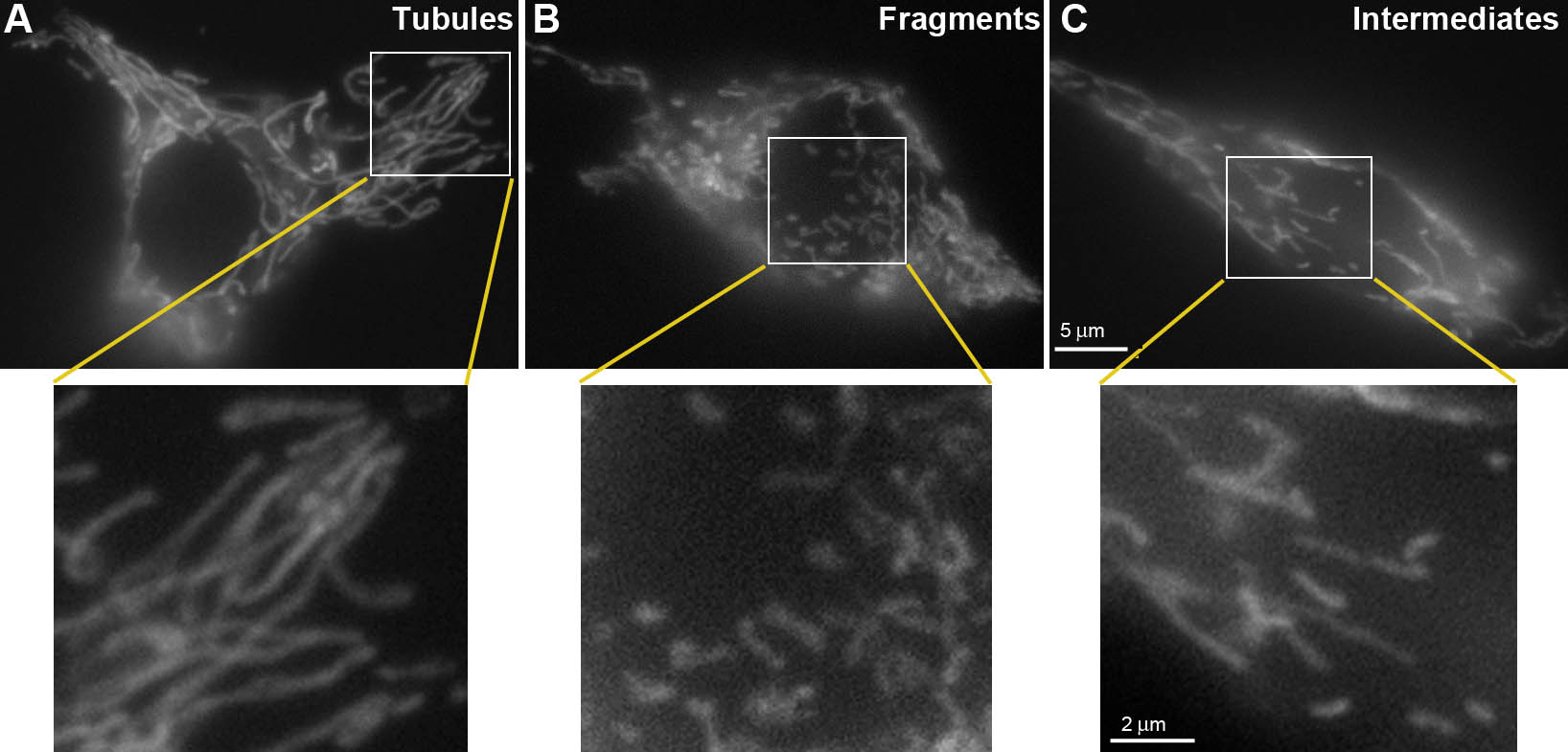

Supplement: Figure S2 — Mitochondrial morphologies categorized in INS-1E cells. INS-1E cells containing long tubules of networks (A; ‘Tubules’), short tubules and small spheres (B; ‘Fragments’), and intermediate tubule length or the mixture of tubules and fragments (C; ‘Intermediates’). Bottom panels are enlarged images of boxed regions. (TIF) [file pone.0060810.s002.tif]

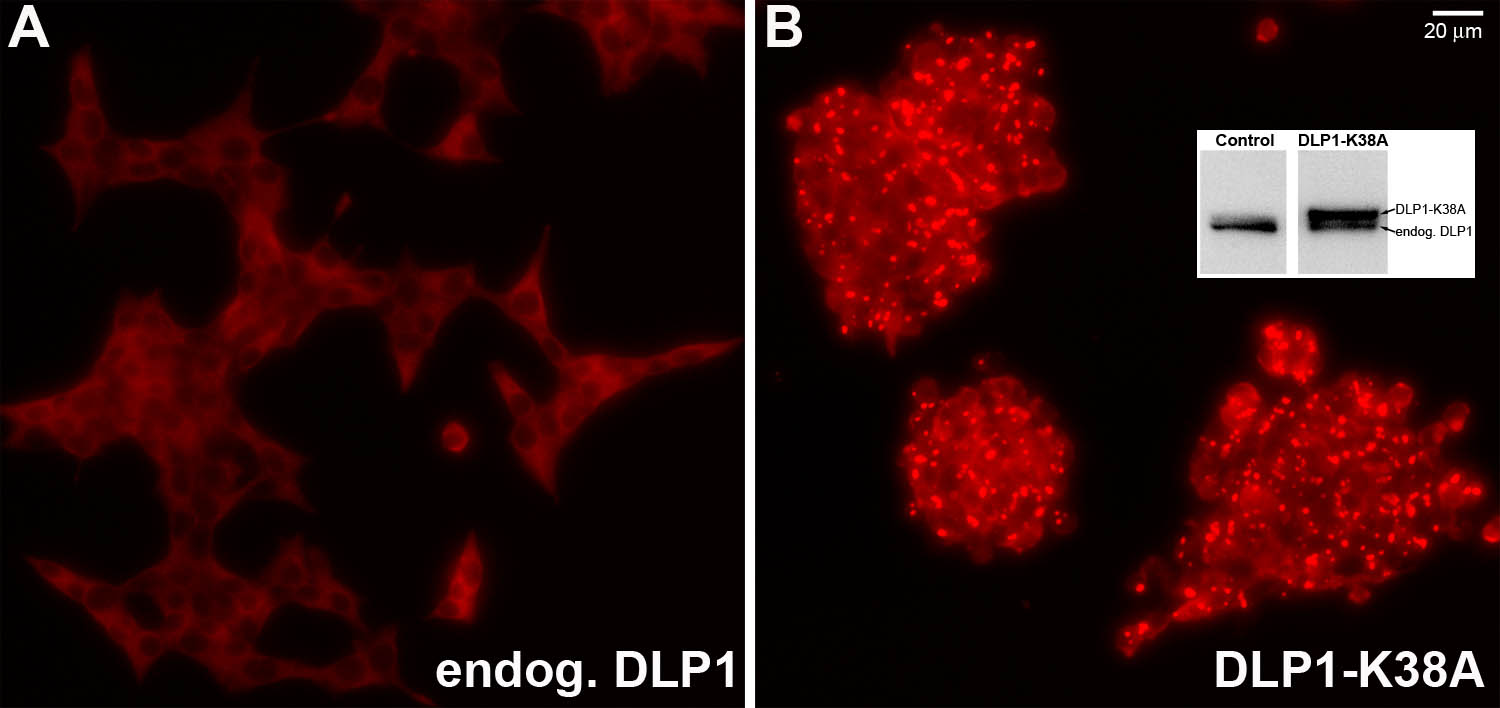

Supplement: Figure S3 — DLP1-K38A expression. The dominant-negative DLP1-K38A mutant was expressed in INS-1E cells for 48 hours by adenoviral infection. Endogenous DLP1 appeared diffuse in the cytosol in this magnification (A). Overexpression of DLP1-K38A induced the formation of bright DLP1-containing aggregates in the cytoplasm (B). Inset shows the DLP1 immunoblot of cell lysates from control and DLP1-K38A-expressing cells. The molecular weight of the overexpressed DLP1-K38A protein is higher due to the use of a larger spliced variant for mutagenesis. (TIF) [file pone.0060810.s003.tif]

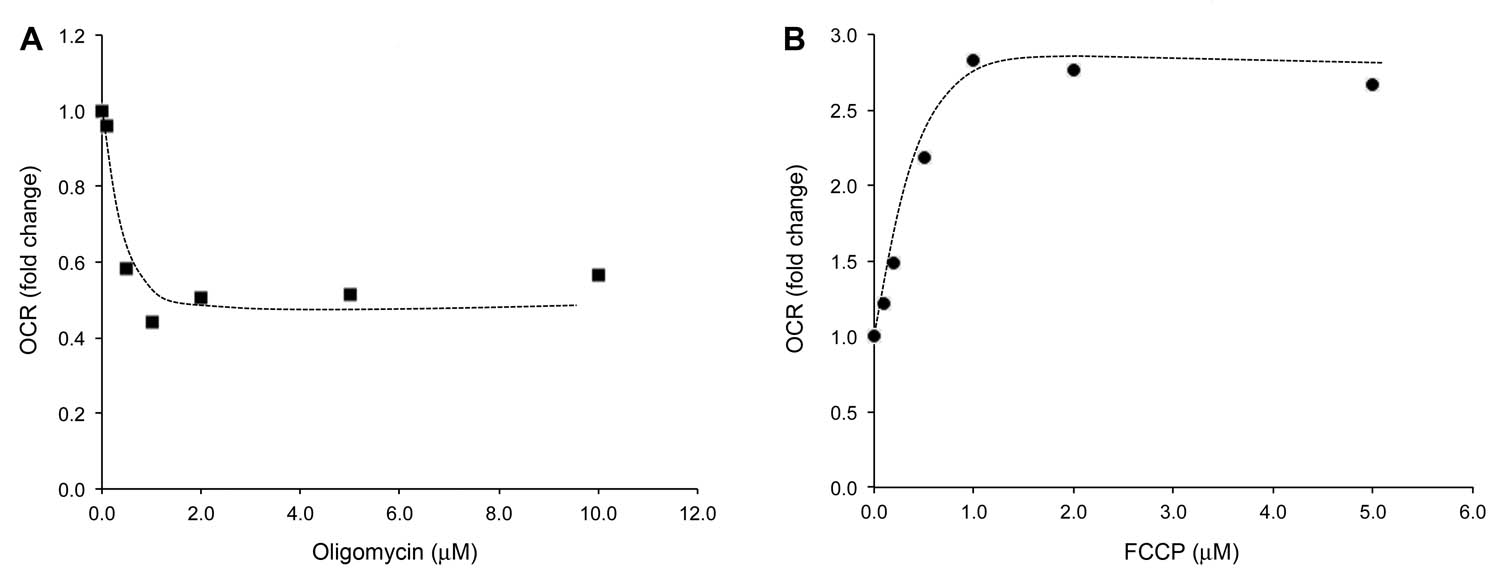

Supplement: Figure S4 — Titration of oligomycin and FCCP. OCR was measured with INS-1E cells in different concentrations of oligomycin (A) and FCCP (B). Concentrations higher than 1–2 µM showed maximal efficacy in both oligomycin and FCCP for decreasing and increasing the OCR, respectively. (TIF) [file pone.0060810.s004.tif]
